# Need for clarifying remote physiologic monitoring reimbursement during the COVID-19 pandemic: a respiratory disease case study

**DOI:** 10.1038/s41746-021-00421-8

**Published:** 2021-03-12

**Authors:** Robert Jarrin, Meredith A. Barrett, Leanne Kaye, Sibel Sayiner, Amanda von Leer, Jennifer Johns, Larissa D’Andrea, Carlos Nunez, Andrey Ostrovsky

**Affiliations:** 1 The Omega Concern, LLC, Washington, DC USA; 2 Department of Emergency Medicine, George Washington University, Washington, DC USA; 3 Department of Biochemistry and Molecular & Cellular Biology, Georgetown University Medical Center, Washington, DC USA; 4 ResMed Science Center, San Diego, CA USA; 5 Propeller Health, San Francisco, CA USA; 6 ResMed, San Diego, CA USA; 7 Social Innovation Ventures, Rockville, MD USA

**Keywords:** Health policy, Respiratory tract diseases

## Abstract

The use of remote monitoring and virtual visits has accelerated to support socially-distanced patient care during the COVID-19 pandemic. Despite the necessity of this expansion, ambiguity in coding is hindering adoption and patient access, most notably for remote physiologic monitoring due to a lack of definition of the term “physiologic”. In this analysis, we describe the history of remote monitoring code development, present several examples in respiratory disease and other chronic conditions in which gaps and confusion remain and suggest ways to clarify and broaden coverage to ensure equitable access to remote monitoring.

## Introduction

The use of remote monitoring to evaluate and manage patients with chronic illness has become even more important in the wake of COVID-19. Remote monitoring, which uses digital health technologies to monitor patient data from anywhere, allows patients and providers to manage the chronic disease without relying on in-person appointments. With remote monitoring, patients can self-manage their condition from home, and providers can leverage data on the patient’s condition to inform their clinical decision-making.

Prior to the COVID-19 pandemic, remote monitoring was gaining traction across several disease states, most notably heart disease, mental health, lung disease, and diabetes. COVID-19 accelerated the adoption of remote monitoring dramatically. With patients unable to see their providers in person, remote monitoring, telemedicine, telehealth, and virtual care saw a 20-fold increase in use^1^, and reimbursement for these services became a focal point for the Centers for Medicare and Medicaid Services (CMS). Remote monitoring is related but distinct from telemedicine by definition and modality in that it involves the interpretation of medical information without a direct, synchronous interaction between the practitioner and beneficiary.

Despite growing interest in the use of remote monitoring, ambiguity^2^ around reimbursement is still creating barriers to adoption^3^ and preventing the delivery of effective, socially distanced care for multiple conditions, which could exacerbate inequitable access to digital tools.

In the past several years, CMS has created multiple Current Procedural Terminology (CPT^®^) codes for remote monitoring services. Four CPT^®^ codes now exist for the provision of “remote physiologic monitoring” services, but CMS has failed to define what it means by “physiologic”, causing providers and payers to question which technologies qualify for reimbursement.

Without clarity on whether a technology qualifies for reimbursement, providers are less able to implement tools that could significantly benefit patient care, lower cost, and increase access for underserved populations. While this is a problem during COVID-19 because of the need for social distancing, it will persist after the pandemic as well.

The current gaps in coding and reimbursement pose a particular problem for the remote treatment of respiratory disease. There are ~16 million people with chronic obstructive pulmonary disease (COPD) in the USA^4^ and just under 25 million with asthma^5^, accounting for $130 billion in direct and indirect costs annually for both COPD^6^ and asthma^7^. Acute and chronic lower respiratory diseases combined are the third leading cause of death^8^ in the USA. Furthermore, the social burden of asthma^9^ and COPD^10^ is demonstrated by the millions of days absent from work and school by both patients and caregivers.

In this analysis, we explore the history and use of remote monitoring codes for reimbursement, present several examples highlighting existing gaps in coding and reimbursement both in respiratory disease and other chronic conditions, and make recommendations for further clarifying and broadening coverage to enhance equitable access to remote monitoring.

## Overview of CPT^®^ and remote monitoring coding

The American Medical Association (AMA) maintains and develops the CPT^®^ code set. CPT^®^ codes are descriptive terms used in reporting medical, surgical, and diagnostic procedures and services that are performed by physicians and other qualified healthcare professionals in the USA. CPT^®^ codes form the basis for reimbursement, research, and tracking medical utilization. Although CPT^®^ codes have existed for numerous remote medical services, until recently, codes to describe general remote monitoring of physiologic data did not exist.

In the 2018 Physician Fee Schedule final rule, CMS concluded that “remote patient monitoring” services could be a significant part of ongoing medical care and that recognition and separate payment for “these services” was to be done “as soon as practicable”.

### CPT^®^ collection and interpretation of physiologic data (i.e., “remote patient monitoring”)

CMS chose to activate, cover, and pay for CPT^®^ Code 99091—a code predicated on collection and interpretation of physiologic data, which CMS referred to as remote patient monitoring. Around the same time, CPT^®^ approved three “remote physiologic monitoring” codes (CPT^®^ 99453, 99454, and 99457), which went into effect on January 1, 2019 (Table 1). In September 2018, CPT^®^ created a fourth “remote physiologic monitoring” code (CPT^®^ 99458) that went into effect on January 1, 2020. It is important to note that these codes represent remote physiological monitoring, but also (and arguably more importantly) treatment management services that go well beyond the review of medical device data. However, CMS still had not defined “physiologic”, as reflected in Table 1.

**Table 1:** Description of current remote monitoring CPT^®^ codes.

Category	CPT^®^ code	In effect	Description	Defines physiologic
Digitally Stored Data Services	99091	Jan. 1, 2018	Collection and interpretation of physiologic data	No
Remote Physiologic Monitoring	99453	Jan. 1, 2019 (2020 CMS proposed valuation for coverage and payment)	Remote monitoring of physiologic parameter(s) (e.g., weight, blood pressure, pulse oximetry, respiratory flow rate), initial; set-up and patient education on the use of equipment	No
	99454	Jan. 1, 2019 (2020 CMS proposed valuation for coverage and payment)	Device(s) supply with daily recording(s) or programmed alert(s) transmission, each 30 days	No
Remote Physiologic Monitoring/Treatment Management Services	99457	Jan. 1, 2019 (2020 CMS proposed valuation for coverage and payment)	Remote physiologic monitoring treatment management services, clinical staff/physician/other qualified healthcare professional time in a calendar month requiring interactive communication with the patient/caregiver during the month; initial 20 min	No
	99458	Jan. 1, 2020 (2019 CMS proposed valuation for coverage and payment)	…; additional 20 min	No

## “Physiologic” limitations: the challenge of reimbursement given general terms

The lack of definition of “physiologic” has created confusion within the reimbursement community, causing providers and payers to question which technologies and physiologic markers qualify for remote “physiologic” monitoring reimbursement, therefore limiting adoption.

The term “physiologic” appears nearly 80 times throughout the AMA CPT^®^ 2020 Professional Edition codebook. The CPT^®^ Editorial Panel publishes the annual codebook to report the rules and guidelines surrounding CPT^®^ codes and descriptors. Despite urgings by stakeholders for clarity, “physiologic” is not formally defined in the CPT® manual, the CPT^®^ Coding Assistant, a coding “Resource and Coverage Guide”, AMA’s Digital Health Implementation Playbook, nor on any associated website.

### CMS coverage and payment of remote physiologic monitoring

In November 2018, CMS extended coverage and payment for CPT^®^ remote physiologic monitoring codes 99453, 99454, and remote physiologic monitoring treatment management services code 99457. In November 2019, CMS announced that it would adopt, cover, and pay CPT^®^ code 99458 for each “additional 20 min”, beginning January 1, 2020. In the Final Rule commentary^11^, CMS acknowledged the need for clarification of the term physiologic and indicated they would consider these issues in future rulemaking. However, as of September 2020, CMS has not issued regulatory, sub-regulatory, or clarifying policy specific to the entire suite of remote physiologic (9945X) monitoring codes. More broadly, the term physiologic does not have a formal definition by CMS within rule, policy, or even in its listing of chronic diseases and associated terms.

## “Physiologic” ambiguity: a respiratory disease case study

There exist a multitude of examples where a narrow definition of physiologic limits the application of remote monitoring, which we describe briefly below. Here, we describe examples in respiratory disease that demonstrate the need for a broadened, clarified definition of physiologic. This respiratory exploration is proposed due to the prevalence and social and economic burden of COPD and asthma, prior published research in this area, provider confusion about coverage, and the relevance of the topic given COVID-19.

### Propeller digital therapeutic

Propeller is a Food and Drug Administration-cleared, digital therapeutic that combines inhaler sensors, companion mobile apps, predictive analytics, and monitoring and feedback to support self-management and clinical care for asthma and COPD^12,13^. Sensors passively monitor the use of inhaled medications, capturing the date and time of each usage, and approximate geographic location (when paired with a smartphone). These signals provide an assessment of daily adherence to controller medication therapy and changes in the use of rescue medications, or short-acting beta-agonists (SABA), which are used for acute relief of asthma and COPD symptoms. Patients have access to these data through web dashboards and smartphone applications. Clinical teams can also access sensor-collected data through a web dashboard to inform clinical decisions such as medication adjustments and receive notifications about patients whose status may be declining.

### SABA use as a physiologic measure

Determination of a patient’s respiratory status is made with a combination of clinical input, including physical examination focused on the work of breathing and chest auscultation, patient-reported outcomes and measures including spirometry, heart rate, respiratory rate, oxygen saturation, and exercise testing. We propose that the signals collected by the inhaler sensor provide an objective assessment of SABA use, providing physiological indicators of stable, worsening, or improving lung health.

SABA use is an important marker of respiratory disease status, specifically level of control, disease burden, and risk^14,15^. Studies have shown that exacerbations—events characterized by a sudden worsening in symptoms requiring an oral corticosteroid, emergency department (ED) visit or hospitalization—may also be identified through increased use of SABA medication. In COPD, SABA overuse is associated with greater disease burden^16^, and upward trends in use have been associated with an increased risk of moderate-to-severe exacerbations^17^. Similar outcomes have been observed in asthma^18^, where increased SABA use has been associated with worsening symptoms and acute exacerbations. Studies have shown that clinically validated asthma and COPD self-reported instruments, such as the Asthma Control Test (ACT)^19^, Asthma Control Questionnaire (ACQ)^20^, and COPD Assessment Test (CAT)^21^, are significantly correlated with SABA use. Lastly, SABA has also been incorporated into the Asthma Medication Ratio^22^, which is a specific HEDIS criteria measure^23^, and is a commonly tracked outcome reported in 68% of studies of exacerbations^24^.

Previously, SABA use could only be captured based on medication pharmacy fill records, patient-reported history, or patients remembering to bring in their medications with a dose counter, making this component of respiratory status require record-keeping and/or presentation to a clinician for assessment. Passive monitoring of SABA with sensors provides a more real-time and objective component of evaluating respiratory status remotely, enabling early intervention to avoid unnecessary exacerbations.

### Clinical benefit of respiratory monitoring

Respiratory digital therapeutics have been studied in multiple randomized controlled trials and observational studies. Across these studies, significant improvements in asthma and COPD outcomes have been demonstrated in association with their use, including reduction in daytime and night-time self-reported symptoms^15^, reduction in SABA use^13,25^, increase in the number of SABA-free days^12,13^, improvement in asthma control as measured by the ACT^26,27^, and improvement in adherence to controller medications^28–31^. These benefits have been observed in diverse participants, including underserved patients with both asthma and COPD^13,26^. Significant reduction in acute healthcare utilization, including ED visits and hospitalization events, has been demonstrated for both asthma and COPD^27,29,32^.

### Ambiguity in multiple disease states

In other disease states, a similar promise yet ambiguity exists. In mental health, codes to support remote cognitive behavioral therapy^33^ are being developed; however, they do not necessarily cover other emerging signals, such as monitoring of smartphone usage or activity patterns, which may provide early indicators of depressive, anxious, or other pathologic behaviors^34^. For Parkinson’s disease, wearable sensors offer promise in remotely monitoring motor fluctuations, immobility, and tremors^35^, which can identify treatable symptoms such as dyskinesia and bradykinesia, but may not be covered by traditional physiologic monitoring codes.

These signals from multiple disease states demonstrate that digital health deployed for remote monitoring can serve as an important tool for medical practitioners, specialists, and clinicians to assist patients living with a multitude of conditions. It is therefore in the interest of those medical professionals who employ the use of remote monitoring technologies to accurately understand medical diagnostic and therapeutic services, particularly in the context of coding, coverage, and payment.

## Regulatory clarity is needed

Without any definition or clarification by AMA/CPT, CMS, or a medical society that formally excludes digital health technologies as a valid mechanism by which to capture physiologic markers for purposes of remote physiologic monitoring, there is no conclusive exclusion to the broad use of the term “physiologic”.

CMS may at any given time produce regulatory or sub-regulatory policy guidance clarifying their position. In the absence of authorities limiting their power, such as national coverage determinations, the contractors in any one of the 12 Medicare Parts A and B and four Durable Medical Equipment Medicare Administrative Contractors regions, acting under their specific geographic jurisdiction, may at their discretion deny claims based on their particular opinion or other arbitrary factors. CPT^®^ code reimbursements are therefore vulnerable given the lack of clear definitions.

As digital health innovation proliferates, CPT^®^ should continue to explore code gaps and create additional remote monitoring codes that describe other ways to evaluate patient status. As announced publicly by CPT^®^ in anticipation of the CPT^®^ Editorial Panel Meeting in October 2020^36^, several applications to create new codes have been submitted for consideration, including one submitted by Propeller Health for Remote Respiratory Status Monitoring Services.

## Conclusion

Based on all of the foregoing, including medical practice and evidence, “physiologic”, as used by CPT^®^ codes for “Remote Physiologic Monitoring” should encompass these services. Inhaler sensors, used in conjunction with medicines and provider services, satisfy the professional and technical requirements^11^ for remote patient monitoring and remote physiologic monitoring CPT^®^ codes.

As the US progresses through the COVID-19 pandemic, remote monitoring and other forms of virtual care will become increasingly critical to delivering effective healthcare while reducing infection risk and patient access to these tools should be equitable. An important step in addressing equity is to ensure that systemic reimbursement barriers, like the ambiguity of the definition of “physiologic”, do not disproportionately affect beneficiaries of public insurance more than those with commercial insurance or who are paying cash. In addition, temporary waivers, such as lifting geographic care site restrictions or administrative sanctions for providers who reduce or waive cost-sharing obligations, could temporarily incentivize providers.

Increased utilization of virtual care in healthcare, which has been implemented on an emergency basis during the COVID-19 pandemic, now needs to be integrated permanently. Chronic respiratory disease care is just one example of the many conditions that could benefit from expanded access to remote monitoring.

## Data Availability

Data in this paper are derived from public resources and published literature.
